# Multidimensional field-based performance profiling in the Latvian national wheelchair basketball team: an exploratory study

**DOI:** 10.3389/fspor.2026.1872790

**Published:** 2026-07-28

**Authors:** Aija Klavina, Gundega Akuratere, Germans Jakubovskis

**Affiliations:** 1 Laboratory of Sports and Nutrition Research, Riga Stradins University, Riga, Latvia; 2 Department of Health Promotion and Rehabilitation, Lithuanian Sport University, Kaunas, Lithuania; 3 Klaipėdos Valstybinė Kolegija/Higher Education Institution, Klaipeda, Lithuania; 4 Latvian Academy of Sport Education, Riga Stradins University, Riga, Latvia

**Keywords:** aerobic capacity, agility, field-based testing, upper-body strength, wheelchair basketball

## Abstract

**Introduction:**

Wheelchair basketball performance depends on multiple factors, including classification, training experience, and sport-specific movement proficiency. This study aimed to evaluate selected physical performance characteristics in wheelchair basketball players of the Latvian national team.

**Methods:**

Nine athletes completed a multidimensional field-based assessments, including the 6-minute push test (6MPT), *T*-test, and upper-body strength assessments and rating of perceived exertion (RPE) following the 6MPT. Descriptive analyses and correlation analyses were performed to examine relationships among performance variables and International Wheelchair Basketball Federation (IWBF) classification points.

**Results:**

The mean distance covered during the 6MPT was 892.6 ± 70.4 m, and the mean *T*-test completion time was 15.17 ± 1.45 s. Dominant handgrip strength averaged 60.0 ± 4.4 kg, while mean RPE following the 6MPT was 5.7 ± 0.7. No significant associations were found between IWBF classification and field-based performance outcomes and considerable inter-individual variability in performance outcomes was observed across functional classes. A significant negative correlation was observed between *T*-test time and 6MPT distance (*r* = −0.781, *p* = 0.013), indicating that athletes with better agility performance tended to cover greater distances during the 6MPT.

**Conclusion:**

Multicomponent field-based testing in this small cohort of Latvian national team wheelchair basketball players demonstrated substantial inter-individual variability in physical performance outcomes. Within the limitations of this heterogeneous sample, no significant associations were observed between IWBF classification and the measured field-based performance variables. Further research involving larger cohorts and broader wheelchair basketball-specific testing protocols is required.

## 1 Introduction

With an estimated 100,000 players across recreational and elite levels, wheelchair basketball ranks among the most widely played Paralympic team sports and is practiced in nearly 100 countries (1). Developing a thorough physical profile is a key component of elite athlete preparation, as the sport simultaneously requires high levels of explosive strength, sprint acceleration, change of direction capacity, and aerobic endurance (2).

In wheelchair basketball, athletes are assigned to classification categories by the International Wheelchair Basketball Federation (IWBF), ranging from 1.0 to 4.5 points, based on trunk stability, balance, and functional movement capacity relevant to wheelchair basketball performance (3, 4). Lower-point players typically exhibit greater physical impairment and reduced trunk control, whereas higher-point players demonstrate greater functional ability. The classification system is designed to minimize the impact of impairment on competition outcomes and promote equitable participation among athletes with diverse disability profiles (5). Despite its central role in competition, the extent to which IWBF classification predicts field-based physical performance remains unclear. This issue is particularly relevant because training-induced adaptations, differences in impairment type, and variability in sport experience may contribute substantially to performance differences among athletes within the same classification category. Although classification level is generally considered an important determinant of sport-specific performance, previous studies have reported considerable within-class variability, suggesting that factors such as training experience, technical proficiency, and physical conditioning may also play a significant role in shaping performance outcomes (6, 7). Consequently, further research is needed to better understand the relationship between classification level and field-based performance measures in wheelchair basketball players.

Physical assessment in wheelchair basketball has traditionally relied on laboratory protocols, particularly cardiopulmonary exercise testing (CPET) using arm crank ergometers. However, these controlled settings do not fully reflect the demands of on-court performance or the manoeuvrability skills required in competition (8). Additionally, differences in work economy and biomechanical patterns between arm crank ergometry and wheelchair propulsion limit the ecological validity of laboratory-based assessments (9, 10). In contrast, field-based assessments conducted on court in the athlete's own sports wheelchair overcome these shortcomings by reproducing the specific movement patterns required in play (4, 8). Importantly, these tests reproduce key movement and propulsion characteristics commonly demonstrated during wheelchair basketball games, including repeated directional changes, sustained wheelchair propulsion, and upper-body force production under competition conditions. The 6-Minute Push Test (6MPT), handgrip dynamometry, and the T-agility test have each demonstrated acceptable levels of reliability and validity in wheelchair sport populations. The 6MPT, in particular, has shown high test-retest reliability and the ability to detect fitness differences among wheelchair users, and represents the most widely validated field-based measure of aerobic capacity across both clinical and athletic wheelchair populations (11). Furthermore, handgrip strength has been shown to correlate with classification level in wheelchair basketball athletes, with higher classified players demonstrating greater grip strength (12). As wheelchair propulsion and sport-specific upper-body demands place considerable stress on grip and forearm musculature, handgrip dynamometry provides a practical and ecologically relevant measure of upper-body strength in this population (13). Additionally, the T-agility test has exhibited good consistency across multiple testing sessions, reflecting the sport's rapid directional changes (7).

A recent systematic review by Petrigna et al. (14) identified only 18 studies that met the criteria for field-based fitness testing in wheelchair basketball, with sample sizes ranging from 8 to 61 athletes and substantial heterogeneity in the test batteries employed. This variability makes it difficult to define standard reference values that are applicable across various national contexts. Moreover, none of the reviewed studies combined portable breath-by-breath metabolic monitoring with court-based field testing. In addition, the existing evidence is predominantly derived from larger, higher-division teams in countries such as Spain, Turkey, and Japan, whereas small, lower-division, and structurally heterogeneous national teams remain substantially underrepresented (14). The present study addresses this gap by applying a standardized and internationally recognized field-test battery within a small national wheelchair basketball program while simultaneously integrating portable metabolic monitoring with court-based performance assessment. The use of a standardized testing protocol also facilitates future multinational data harmonization and pooled analyses, an approach increasingly recognized as essential for overcoming the persistent sample-size limitations that characterize Paralympic sport science research (14, 15). In Latvia, wheelchair basketball players have been routinely evaluated using laboratory-based cardiopulmonary exercise testing (CPET), whereas court-based performance assessments have been reported only once, in a study involving eight Latvian athletes (6). This indicates that the Latvian national wheelchair basketball team has consisted of a limited number of athletes for many years. Moreover, the team is both small and heterogeneous, with players differing in classification levels, impairment type, and wheelchair basketball experience, all of which are factors known to influence physical performance outcomes (6, 12). Under these conditions, an individual profiling strategy is more appropriate than group-level comparisons, which, in a small and heterogeneous sample, may obscure important between athlete differences. Correlation analyses were included primarily to explore potential performance-related patterns within this cohort that may generate testable hypotheses for future adequately powered investigations.

The aims of this study were twofold. First, we aimed to investigate whether IWBF classification is associated with field-based performance outcomes in a small, structurally heterogeneous national cohort, to examine the inter-relationship between agility and aerobic endurance performance, and to demonstrate the feasibility of combining portable metabolic monitoring with standardized court-based field testing within national training camp constraints. The secondary aim of this study was to evaluate aerobic performance, agility, and upper-body strength in wheelchair basketball players using field-based tests, and to examine the relationships between performance outcomes, physiological responses, and classification level. It was hypothesized that substantial inter-individual variability would be observed, and that the classification level alone would not fully explain field-based performance outcomes within this heterogeneous national team cohort. In this study, an individualized profiling approach refers to the multidimensional evaluation of each athlete across several complementary performance domains, including aerobic endurance, physiological response, agility, upper-body strength, perceived exertion, and classification level. This approach may be particularly informative in small and heterogeneous wheelchair basketball cohorts, where group-level analyses alone may mask meaningful athlete-specific differences.

## 2 Materials and methods

### 2.1 Participants

Nine male wheelchair basketball athletes (A) from the national team of Latvia (B division), who were participating in a national training camp, were included in this study. Participants reported varying training schedules outside the camp period, ranging from 2 to 7 sessions per week. The sport experience in wheelchair basketball varied from 1 up to 22 years. All participants and the team coach were informed about the purpose of the study. The study protocol was developed in accordance with the Declaration of Helsinki and approved by the Ethics Committee of the Latvian Academy of Sport Education (No. 1/2023-29-09). Each athlete provided written informed consent prior to participation. The health condition of athletes was as follows: spinal cord injury (*n* = 5), spina bifida (*n* = 1), lower limb amputations (*n* = 1), cerebral palsy (*n* = 2). The sample included athletes across a wide classification range (1.5–4.5 points), reflecting heterogeneity in functional ability. According to the IWBF classification system, lower-point athletes generally present greater activity limitation and trunk impairment, whereas higher-point athletes demonstrate greater functional capacity during wheelchair basketball performance. For individual-level analyses, athletes were assigned the following codes: A1 (1.5), A2 (2.0), A3 (1.5), A4 (2.0), A5 (1.5), A6 (3.5), A7 (3.5), A8 (3.0), and A9 (4.5). All athletes completed field-based tests, including the 6MPT, *T*-test, and handgrip dynamometry. These tests were selected because they evaluate key physical components relevant to wheelchair basketball performance, including aerobic endurance, ability to change direction, and upper-body strength. The assessment was conducted on a basketball court using the wheelchairs typically employed during games. All tests and measurements were performed in the morning, separate from scheduled training sessions.

### 2.2 Methods

#### 2.2.1 6 Min push test (6-MPT)

Cardiovascular endurance was evaluated using the 6MPT, a field-based assessment of functional capacity adapted from the Six-Minute Walk Test (6MWT) for individuals who use a wheelchair (11). The protocol was conducted in strict accordance with the guidelines published by the National Center on Health, Physical Activity and Disability (NCHPAD) and the American Thoracic Society (ATS). Breath-by-breath ventilatory and gas-exchange variables were recorded continuously throughout the test using the Cortex Metamax 3B portable metabolic analyser (Cortex Biophysik GmbH, Leipzig, Germany), worn by each participant in a lightweight harness. Heart rate was monitored continuously via a Polar chest strap (Polar Electro Oy, Kempele, Finland) integrated with the Cortex system. Prior to testing, participants rested for five minutes in proximity to the starting point; resting heart rate was recorded before the test start.

The test was performed on a flat indoor basketball court surface to ensure unbiased, consistent rolling resistance and conditions across all participants. A flat, straight 15-metre course, marked at each end by a cone, formed a 30-metre loop. All athletes trained and competed in their own personal sports wheelchairs, which were individually configured to their specific physical and functional requirements. Participants were instructed to propel as fast as possible during the six-minute period, pivoting briskly around each cone. To maximize procedural consistency and reproducibility, standardized verbal encouragement was delivered by the same examiner at fixed time points in strict accordance at minutes 1, 2, 3, 4, 5, and at 5 min 30 s using identical wording for all participants. Completed laps were counted by the examiner using a lap counter, and the remaining partial distance at the 6-minute endpoint was measured with a measuring tape. Total distance covered (m) was calculated as the primary outcome.

#### 2.2.2 *T*-test

**Figure 1:**
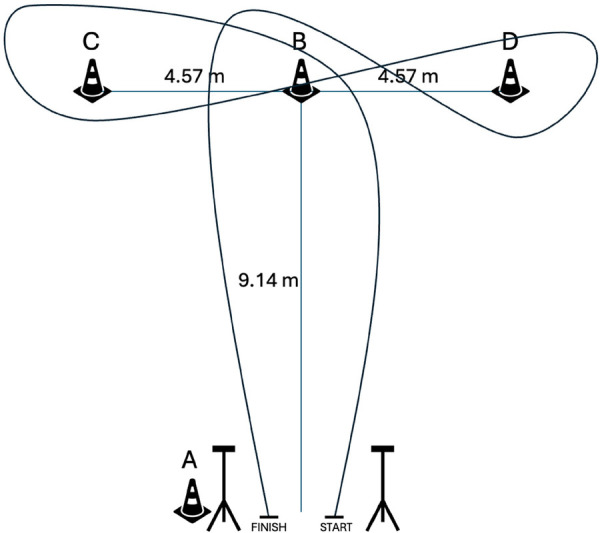
*T*-test.

Wheelchair agility was assessed using a modified version of the standardized *T*-agility test, which has demonstrated acceptable levels of reliability and validity in wheelchair basketball populations (7). The test layout was configured on a flat indoor basketball court using four cones arranged in a T-shape: cone A was positioned at the start/finish line, cone B was placed 9.14 m directly ahead, and cones C and D were set 4.57 m to the left and right of cone B, respectively (Figure 1). Performance times were recorded to the nearest 0.01 s using the Microgate Racetime 2 electronic timing system (Microgate S.r.l., Bolzano, Italy) with infrared photocell gates positioned at cone A to eliminate manual measurement error. Upon the start signal, participants propelled forward from the start line toward cone B, looped around it, then propelled laterally to cone D, looped around it, then propelled across the full width of the course to cone C, looped around it, and finally returned through cone B to cross the finish line at cone A. Athletes propelled in a forward-facing direction throughout the entire course. Participants were required to touch the top or base of each cone with their hand during the trial. Trials were immediately disqualified and repeated if a participant knocked over a cone, failed to make physical contact, or deviated from the prescribed movement sequence. Two practice trials were permitted prior to recorded attempts. Each athlete completed two maximal trials with a minimum three-minute rest interval between attempts, and the fastest completed time was retained for analysis.

#### 2.2.3 Grip strength

Grip strength was assessed using a hydraulic handgrip dynamometer (MSD Europe, Model SH5001), with the handle position standardized at the second setting for all participants to ensure an optimal grip span and strict replicability of measurements across the cohort. During the measurements, participants were seated in their own sports wheelchairs with the shoulder in a neutral position, the elbow flexed at 90°, the forearm in a neutral position, and the wrist positioned at approximately 30° of extension and 10° of ulnar deviation, adhering to the standardized positioning guidelines established by the American Society of Hand Therapists (16). Each athlete completed two maximal trials per hand, and the highest value obtained from the dominant hand was used for analysis. This approach was adopted to provide a standardized measure of maximal upper-body strength that is comparable with previous wheelchair basketball studies and to minimize the influence of impairment-related upper-limb asymmetries arising from the heterogeneous neurological and musculoskeletal characteristics of the study cohort (12). Participants were instructed to squeeze the dynamometer maximally for 3 s and then relax. A 30-s rest interval was provided between successive trials to minimize muscle fatigue and ensure baseline recovery (13).

#### 2.2.4 Subjective exertion assessment

The modified Borg Perceived Rate of Exertion (RPE) scale was used at the end of the 6MPT, with participants being asked about their perceived physical effort during the test (23, 24). Rating of perceived exertion (RPE) is a subjective measure based on individual perceptions of physical effort, including sensations such as breathing rate, heart rate, perspiration, and muscle fatigue. The scale ranges from 0 (rest) to 10 (maximal exertion), with verbal anchors of 1 (very easy), 3 (moderate), 5 (hard), and 7 (very hard).

### 2.3 Statistical analyses

All statistical analyses were performed using IBM SPSS Statistics for Windows, version 31.0 (IBM Corp., Armonk, NY, USA). Descriptive statistics are presented as mean (standard deviation, SD), with minimum and maximum values reported where appropriate. In addition to descriptive group statistics, an individualized profiling approach was applied whereby each athlete's performance outcomes were examined across all measured domains. Individual profiles were compared descriptively with group averages and with athletes of similar IWBF classification to explore within-class variability and identify performance patterns that could not be adequately captured through group-level analyses alone.

The normality of data distribution was assessed using the Shapiro–Wilk test. Given the small sample size (*n* = 9), heterogeneous participant characteristics, and exploratory nature of the study, non-parametric statistical procedures were used for the primary association analyses. Spearman's rank correlation coefficients were calculated as exploratory analyses to examine potential relationships between field-based performance variables (6-min push test distance, *T*-test time, and handgrip strength), physiological responses (VO_2_), perceived exertion (RPE), and IWBF classification points. Given the exploratory nature of the study and the limited sample size, correlation analyses were interpreted descriptively and used primarily to explore potential associations between variables. Correlation strength was interpreted as trivial (<0.1), small (0.1–0.3), moderate (0.3–0.5), large (0.5–0.7), and very large (>0.7) (17). Statistical significance was set at *p* < 0.05 for all analyses.

## 3 Results

Descriptive characteristics and individualized performance profiles of the athletes are presented in Tables 1, 2. Table 2 was specifically designed to present exploratory comparison of inter-individual physiological and performance characteristics across participants. The mean age of the athletes was 37.89 (SD = 6.13) years, with a mean body mass of 72.72 (SD = 12.03) kg.

**Table 1:** Participant characteristics and outcome measures.

Descriptive statistics	Mean	Std. deviation	Minimum	Maximum
Age	37.89	6.13	28.0	45.0
Body Weight (kg)	72.72	12.03	55.0	90.0
6-MPT				
Total distance (m)	892.56	70.40	776	991
Total laps	29.44	2.45	25	33
VO_2_ peak_abs (L/min)	1.88	.30	1.55	2.65
VO_2_ peak_relat (mL/min/kg)	27.02	4.96	19.37	31.44
Max HR (bpm)	165.67	11.43	141	178
RER (Max)	1.07	.090	.92	1.21
VE (l/min)	94.18	13.40	68.88	107.67
RPE (Borg)	5.67	.71	5	7
*T*-test				
Time (s)	15.17	1.45	14.10	18.60
Grip strength				
Dominant hand (kg)	60.00	4.44	54	67

**Table 2:** Individual sport experience and results of field-based performance.

Athlete	IWBF functional class	Training in W/basket-ball (years)	Training sessions per week	Training volume (h/week)	*T* test (s)	Hand grip strength (kg)	Distance (m)	VO_2_ mean (L/min)	VO_2_ per minute (L/min)					
									1	2	3	4	5	6
A1	1.5	21	6	2	18.60	57	776.0	1.91	1.32	1.80	1.77	2.23	2.05	2.08
A2	2.0	15	4	1	15.20	62	873.0	1.55	1.55	1.32	1.45	1.36	1.49	2.08
A3	1.5	7	4	1	14.30	61	885.0	1.92	1.15	1.42	1.92	2.28	2.17	2.27
A4	2.0	20	7	1	14.20	64	946.0	1.84	1.58	1.78	1.81	1.91	2.04	1.93
A5	1.5	18	4	2	14.30	63	966.0	1.73	1.15	1.77	1.81	1.83	1.78	1.88
A6	3.5	1	3	2	15.10	67	915.0	2.65	1.85	2.87	2.88	2.90	2.74	2.64
A7	3.5	11	2	2	16.20	55	810.0	1.73	1.19	1.95	1.95	1.85	1.83	1.55
A8	3.0	22	3	2	14.50	54	990.6	1.85	1.43	1.97	1.87	2.01	1.90	1.81
A9	4.5	18	1	1	14.10	57	871.4	1.81	1.25	1.74	2.22	2.3	1.78	1.44

### 3.1 6-MPT results

**Figure 2:**
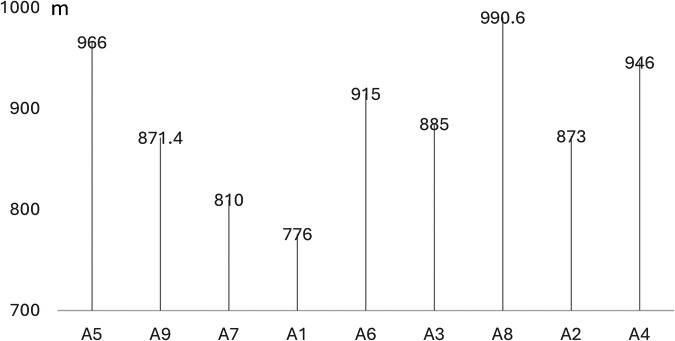
Individual results of total distance completed in 6MPT. A(athlete): points (functional classification) A1:1.5; A2:2.0; A3:1.5; A4:2.0; A5:1.5; A6:3.5; A7:3.5; A8:3.0; A9:4.5.

**Figure 3:**
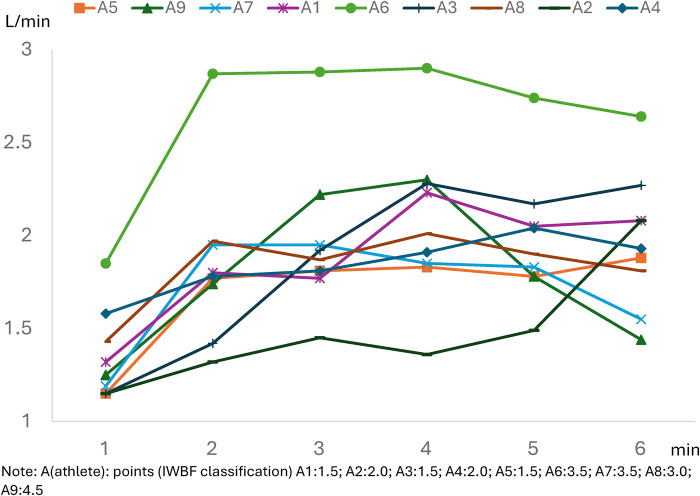
Minute-by-minute oxygen uptake (VO_2_) results during the 6MPT.

The mean distance covered during the 6MPT was 892.56 (SD = 70.40) m. No significant relationship was observed between IWBF classification and 6MPT performance (*p* > 0.05). When analyzed at the individual level (Table 2, Figures 2, 3) the distribution of performances across IWBF classes (1.5–4.5 points) did not reveal a clear trend of increasing distance with higher classification category. Notably, a low-point player (A5; 1.5) demonstrated one of the highest distances (966 m), whereas a high-point player (A9; 4.5) achieved only moderate performance (871 m).

Furthermore, analysis of VO_2_ during the 6MPT revealed distinct physiological response patterns among athletes. Overall, VO_2_ increased rapidly during the first 2–3 min of the test, followed by a plateau or slight decline toward the final stages, indicating attainment of near steady-state aerobic metabolism (Figure 3). Athletes with higher IWBF classifications (A9: 4.5 points; A6: 3.5 points) generally demonstrated higher VO_2_ values throughout the test and reached peak values earlier, which may reflect differences in aerobic responses and wheelchair propulsion characteristics between athletes. For example, A6 exhibited a rapid increase in VO_2_, peaking already second minute (∼2.87 L/min) and maintained it till minute 4 with slight decline towards end of the test. In contrast, lower-class athletes (e.g., A1, A3, A5—all 1.5 points) showed lower absolute VO_2_ values and a more gradual increase across the test duration. However, some variability was observed within this group, with A3 demonstrating relatively high VO_2_ values at the end of the test, indicating individual differences in conditioning. Regarding RPE most participants reported values between 5 and 6, indicating relatively consistent perceived effort across the group despite variability in performance outcomes.

### 3.2 *T*-test results

**Figure 4:**
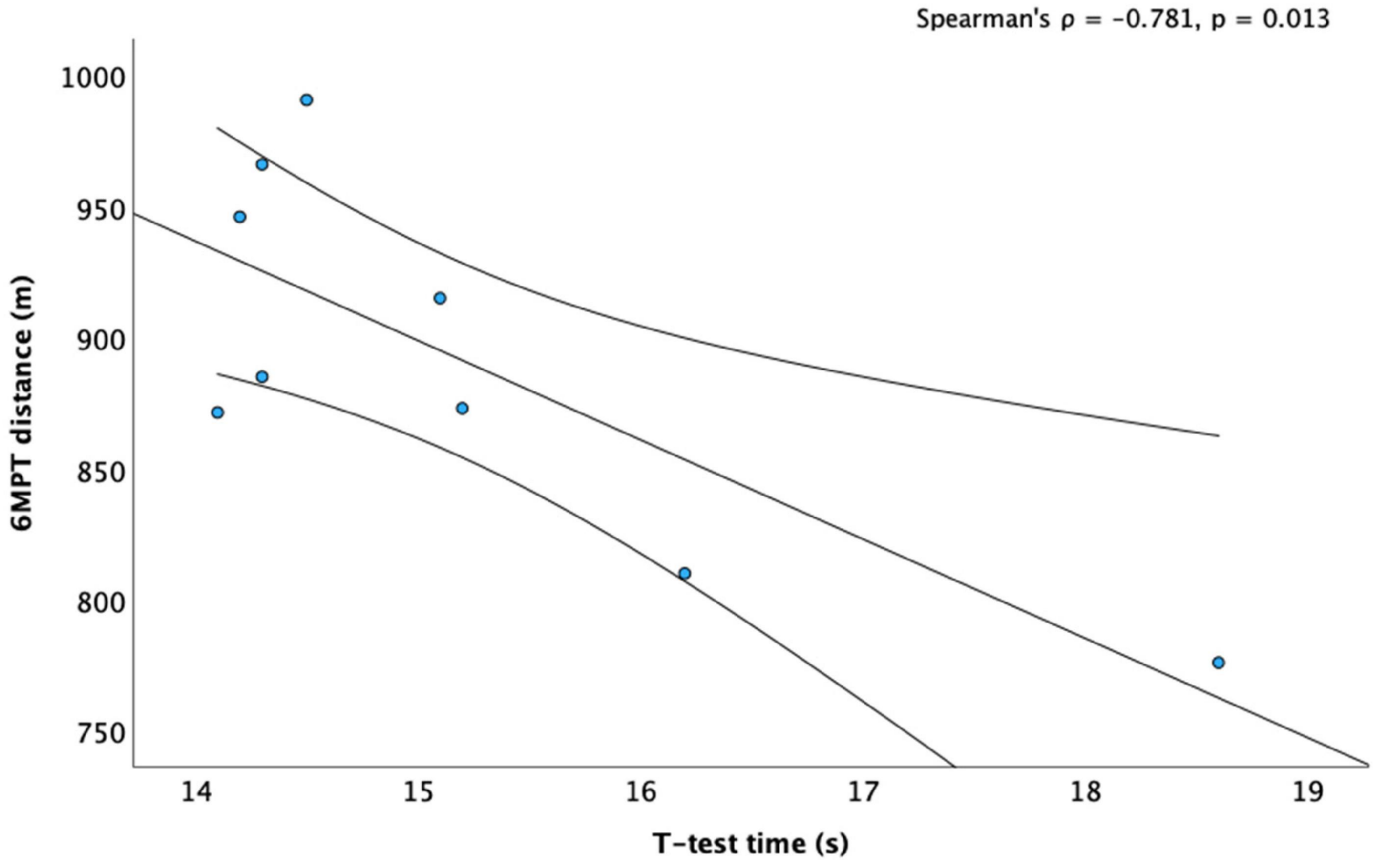
Correlations between 6MPT and *T*-test results.

The mean *T*-test completion time was 15.17 (SD = 1.45) s, with individual performances ranging from 14.10 s to 18.60 s. At the individual level, the fastest times were recorded by A9 (4.5 points; 14.10 s), A4 (2.0 points; 14.20 s), closely followed by A5 and A3 (both 1.5 points; 14.30 s). Notably, agility performance did not follow a clear trend across functional classification levels. However, correlation analysis (Table 3, Figure 4) revealed a significant negative association between *T*-test performance and 6MPT distance (*r* = −0.781, *p* = 0.013), indicating that better agility performance (lower *T*-test time) was associated with greater distance covered during the 6-minute test.

**Table 3:** Spearman's rank correlations between field-based performance variables and IWBF class points.

Variable	IWBF (points)	6MPT (m)	Hand grip (kg)	*T*-test (s)
IWBF (points)	1	−0.032 (−.696, .584)	−0.281 (.357, −.872)	−0.213 (−.667, .811)
*p*-value		0.935	0.464	0.581
6MPT (m)	−0.032 (−.696, .584)	1	0.312 (−.521, .904)	−.781* (−.943, −.029)
*p*-value	0.935		0.413	0.013
Hand grip (kg)	−0.281 (.357, −.872)	0.312 (−.521, .904)	1	−0.31 (−.786, .575)
*p*-value	0.464	0.413		0.417
*T*-test (s)	−0.213 (−.667, .811)	−.781* (−.943, −.029)	−0.31 (−.786, .575)	1
*p*-value	0.581	0.013	0.417	

*r* (CI 95%).

* *p* < 0.05.

### 3.3 Handgrip strength (dominant hand)

The mean dominant handgrip strength was 60.00 (SD = 4.44) kg, with individual values ranging from 54 kg to 67 kg. The highest grip strength values were recorded in A6 (3.5 points; 67 kg), A4 (2.0 points; 64 kg), and A5 (1.5 points; 63 kg). Conversely, the lowest values were observed in A8 (3.0 points; 54 kg) and A7 (3.5 points; 55 kg). Similar to agility performance, handgrip strength did not show a clear association with IWBF classification.

## 4 Discussion

The present study provided a multidimensional field-based assessment of physical performance in wheelchair basketball players of the Latvian national team, integrating performance, physiological, and perceptual measures within an individualized profiling framework.

From a methodological perspective, the integration of portable breath-by-breath metabolic monitoring with standardized court-based field testing proved feasible within the operational constraints of a national team training camp. All assessments were completed during a single morning testing session without disrupting the scheduled training programme, and valid VO_2_ data were successfully obtained from all participants. The Cortex Metamax 3B portable metabolic system was well tolerated by the athletes during the 6MPT, and no technical difficulties or data acquisition failures were encountered. These findings support the practical applicability of combining portable metabolic monitoring with field-based performance testing in wheelchair basketball and demonstrate that physiologically informed athlete assessment can be conducted outside a laboratory environment.

Due to the limited number of participants in this study the individualized profiling approach provided additional insight beyond group-level summary statistics. The main findings descriptively suggest that no significant associations between classification category and field-based performance outcomes were observed within this cohort. For example, athletes with similar classification scores demonstrated markedly different endurance, agility, strength, and physiological response profiles. These observations suggest that athlete-specific factors, including training background, conditioning status, and wheelchair sport experience, may contribute importantly to performance outcomes. Consequently, individualized profiling may represent a useful complementary approach for athlete monitoring in small and heterogeneous wheelchair basketball programs where traditional group comparisons are inherently limited.

The average distance covered during the 6-minute push test in this study was comparable to values previously reported in second-league wheelchair basketball players from Turkey, but lower than those observed in first-league athletes (18). Previous research has demonstrated that playing experience significantly influences physical fitness, with a positive association between years of participation in wheelchair basketball and performance in specific skill tests (19). A similar descriptive trend was observed in this study where athletes with greater wheelchair basketball experience within the same classification group tended to demonstrate superior performance outcomes. However, these observations should be interpreted cautiously, as training experience was not formally incorporated into inferential statistical analyses. Furthermore, although athletes with higher IWBF classification points tended to reach higher absolute VO_2_ values, this did not consistently present superior performance. This observation aligns with previous research in wheelchair basketball players (7), which suggests that besides physiological capacity performance depends also on mechanical efficiency and technical skill. In the present study, some lower-class athletes completed longer distances despite lower VO_2_ values, suggesting that propulsion efficiency, pacing strategies, and previous wheelchair sport experience may also influence field-based performance outcomes. The relatively homogeneous RPE values observed across participants are in agreement with previous findings (20) indicating that trained wheelchair basketball players are capable of regulating effort to maintain a reliable perception of exertion. However, RPE-based training loads provide different information than objectively measured fitness outcomes and should therefore be used as a complement to other objective methods.

The average *T*-test performance in this study was comparable to values previously reported in Spain (7), and in female wheelchair basketball players in Japan (21). Furthermore, no significant relationships between IWBF classification and field-based performance outcomes were observed within this small cohort of national team athletes. However, these findings should be interpreted cautiously due to the small sample size and unequal representation across classification levels, with some classes including only one or two athletes. Considerable inter-individual variability was observed across performance measures, suggesting that factors beyond functional may contribute to performance outcomes. Previous research has proposed that wheelchair agility is influenced not only by functional capacity but also by propulsion technique, chair-handling skills, and neuromuscular coordination developed through sport-specific training (4). The variability observed within classification levels in this study is consistent with this perspective and may reflect differences in training experience, physical conditioning, and technical proficiency among athletes.

A notable finding was the strong negative correlation between *T*-test time and 6MPT distance, indicating that athletes with better agility performance tended to cover greater distances during the endurance test. Although the cross-sectional design does not allow causal inferences, this relationship suggests that agility and endurance-related performance characteristics may be closely linked in wheelchair basketball. Similar observations have been reported in previous studies demonstrating that components of physical fitness in wheelchair basketball are interrelated rather than independent (7, 21). From a practical perspective, this integrated performance structure suggests that training approaches targeting aerobic and neuromuscular capacities simultaneously rather than in isolation, may be most beneficial for wheelchair basketball athletes. However, given the exploratory nature of this analysis and the number of pairwise comparisons examined, this finding should be regarded as hypothesis-generating and requires confirmation in larger, adequately powered investigations.

Handgrip strength values demonstrated moderate variability and were not associated with classification level. Both the average and individual results observed in the present study were slightly higher than in previously reported (7). In line with previous reports, upper-body strength more depends on training exposure and sport-specific demands than by classification level alone (22). While handgrip dynamometry provides a practical assessment of strength, it may not fully capture the complex, multi-joint nature of wheelchair propulsion. This observation is supported by the physiological demands of wheelchair basketball, as handgrip strength is primarily developed through the chronic mechanical loading of the wrist flexors, intrinsic hand muscles, and forearm musculature during repeated push-rim propulsion, wheelchair maneuvering, and ball-handling activities (13). Consequently, the strength adaptations are likely driven by accumulated sport-specific training and playing experience rather than impairment classification alone, which may explain the substantial variability observed within classification levels in the present cohort. Therefore, future studies should consider incorporating more sport-specific strength assessments.

Overall, the present findings suggest that physical performance outcomes in this national cohort may be influenced by multiple interacting factors beyond classification alone, including training experience, propulsion technique, and sport-specific conditioning. However, given the limited sample size and unequal distribution across classification levels in this study, the findings should be interpreted cautiously and cannot be generalized to the broader wheelchair basketball population. Importantly, the present study demonstrates the practical feasibility of implementing individualized multidimensional field-based athlete monitoring within a small national wheelchair basketball program, thereby contributing preliminary evidence for sport-specific performance assessment approaches in wheelchair basketball.

### 4.1 Limitations and future directions

The present study has several limitations that should be acknowledged. First, the small sample size and unequal representation across IWBF classification levels limited the statistical power and reduced the ability to detect potential relationships between classification level and performance outcomes. Given the exploratory nature of the study and the heterogeneity of the sample, the findings should be interpreted cautiously and cannot be generalized beyond this cohort of Latvian national team athletes.

Second, although the selected field-based tests provided valuable information regarding endurance, agility, upper-body strength, and perceived exertion, the testing battery did not include several important wheelchair basketball performance components, such as sprint performance, propulsion biomechanics, or sport-specific technical skills. Therefore, the present assessment reflects only selected components of physical performance. Additionally, RPE was assessed only following the 6MPT and was not analyzed in relation to objective physiological measures such as VO_2_ or heart rate, which limited the interpretation of perceived exertion in relation to objective performance outcomes. Third, differences in sport experience, training frequency and volume, and impairment type may have contributed to the observed inter-individual variability. Additionally, prior familiarity with field-based wheelchair propulsion tests and pacing strategies may have influenced individual performance and physiological responses during the assessments. Finally, due to the cross-sectional design, causal relationships between training background, classification category, and performance outcomes cannot be established. Furthermore, individual wheelchair configuration parameters such as wheel size, seat height were not systematically recorded. While the use of each athlete's personal sport wheelchair enhances ecological validity and reflects real competition conditions, the absence of equipment data limits the ability to fully account for potential configuration-related performance differences between athletes.

Despite the limited generalizability of the present findings, the selected field-based assessment approach may have practical applications for individualized athlete monitoring in other wheelchair sports involving wheelchair propulsion and upper-body performance demands, such as wheelchair rugby or wheelchair tennis. However, sport-specific movement patterns and physiological requirements differ across disciplines, therefore, further validation of these testing procedures in other wheelchair sport populations is necessary.

### 4.2 Conclusion

The selected multidimensional field-based testing in this small cohort of Latvian national team wheelchair basketball players demonstrated substantial inter-individual variability in physical performance outcomes. Within the limitations of this small and heterogeneous sample, no significant associations were observed between IWBF classification and the measured field-based performance variables. The present exploratory findings indicate that individual conditioning, training background, and sport-specific movement proficiency may contribute importantly to wheelchair basketball performance and should be further investigated in larger cohorts.

## Data Availability

The raw data supporting the conclusions of this article will be made available by the authors, without undue reservation.
